# Rewords and Struggle of Online Teaching of Pathology During the COVID-19 Pandemic: Middle East Medical Students’ Judgment

**DOI:** 10.7759/cureus.33377

**Published:** 2023-01-04

**Authors:** Omer Abdelbagi

**Affiliations:** 1 Department of Pathology, Umm Al-Qura University, Al-Qunfudhah, SAU

**Keywords:** medical school students, student perception, online, pathology, covid-19

## Abstract

Introduction: Online learning involves the delivery of educational instructions regarding a subject using the internet. Pathology is an experimental subject that requires students to learn about disease development via unpleasant photos and slides. This study aims to determine the rewards and struggles of online pathology learning during the coronavirus disease 2019 (COVID-19) pandemic at the Al-Qunfudah Medical College, Kingdom of Saudi Arabia.

Methods: Using an online questionnaire comprising three sections of 23 questions (12 questions about the positive perception of online teaching and 11 about the negative perception), we surveyed second and third-year pathology students about their perception of online education. The positive and negative perceptions of the students were compared using the chi-square test (p < 0.05).

Results: About 77% of the students (n = 85/110) responded to the survey. Female students (n = 43, 50.6%) had a significantly higher positive perception of online learning (p < 0.001); male students (n = 42, 49.4%) had a high negative perception of online teaching (p < 0.035). Nearly 70% of the students agreed that the Blackboard platform (Anthology Inc., Boca Raton, FL) made learning easy. About two-thirds of students agreed that the pre-lecture video produced by the teachers, when shared before the lesson, made the pathology lectures easy.

Conclusion: Female students were more favorable toward online pathology learning. Extensive training provided to teachers can significantly increase the support given to students during online teaching.

## Introduction

Online learning comprises educational instructions delivered in an environment powered by the internet for teaching subject matter via an upgraded combination of synchronous and asynchronous learning approaches [1]. Online learning exercises are not inferior to conventional learning activities in this virtual era. Well-planned online higher education should be reasonably accessible, receptive, and resilient to accommodate changing times in learning methods [2]. The recent coronavirus disease 2019 (COVID-19) pandemic allowed the full strength of the online teaching network to be utilized to deliver online courses. Many universities and colleges worldwide have used this mode of education so far, and should further enhance collaboration and share facilities to create a worldwide academic web [3].

Pathology is an application-based subject that correlates the interaction between a host and the etiology of the diseases affecting it (pathogenesis) [4]. It is a mandatory subject in almost all medical programs to bridge the gap between basic sciences and clinical skills, and at the Al-Qunfudah Medical College, Umm Al-Qura University, Makkah, Saudi Arabia, pathologists teach the subject through offline lectures, e-learning, and task-based learning; therefore, tutors are more facilitators than simply information providers [5]. However, the pandemic forced many institutions to adopt online teaching methods instead of physical learning (synchronous) to reduce the risk of COVID-19 infection in addition to reducing the psychological burden of traveling during the pandemic [6].

Many faculties primarily faced two challenges while switching to online teaching: firstly, which platform to use, and second, all students must have sufficient internet access and speed to support all displayed activities [7]. Nevertheless, it was possible to teach pathology online during the pandemic by utilizing available virtual methods, such as learning platforms e.g. Blackboard (Anthology Inc., Boca Raton, FL) [8]. This novel learning experience during the COVID-19 pandemic could serve as a paradigm for future pathology virtual teaching efforts in medical science education [9].

It is known that the pathology course warrants learning through relatively unpleasant photos and slides that must be viewed in high resolution to understand the underlying changes; therefore, students studying pathology should be offered sincere technical support for online learning [10]. Furthermore, recent challenging circumstances have subjected students to a great deal of psychological stress. Despite having considerable knowledge of disease prevention and control, their quality of life has been greatly affected by avoiding public gatherings; even well-informed medical students need psychological help [11]. COVID-19 significantly affected students’ mental and general health, well-being, education, and comprehension [12]. On the other hand, the pandemic-era online teaching experience could be used to meet the needs of students who faced non-pandemic-related difficulties in studying on-site pathology education, such as family constraints, disability, and economic challenges [13].

Many benefits, such as enhanced knowledge gain and greater memorization, have been described for online lectures more than for conventional face-to-face learning and overall student marks in pathology were higher during online learning compared to the previous offline semesters [14].

This study aims to determine the rewards and struggles of online teaching of pathology during the COVID-19 pandemic at Al-Qunfudah Medical College, Kingdom of Saudi Arabia.

## Materials and methods

This was a cross-sectional study. The sampling technique chosen for conducting this study was a non-random selected sample comprising the Al-Qunfudah Faculty of Medicine students, a model for all medical students in Saudi Arabia. Second and third-year pathology students were recruited. The second-year curriculum involves general pathology, including cell injury, inflammation and healing, hemodynamics, and neoplasia. In the third year, the students are taught systemic pathology, integrated into system-based courses in our university setting. Umm Al-Qura University adopted the Blackboard network (founded by the University of Botswana in 2002) as a platform for online teaching. Since then, the network has been widely accepted by universities and colleges globally, with a wide range of applications [15]. Since all our students speak Arabic as their first language, and there are no international students in our faculty, we translated the questions to Arabic and back to English, as mentioned in a previous study [16], to secure certainty. All students (males and females) from the second and third years were included in the study. Exclusion criteria included those who failed to complete the consent and those from the first, fourth, fifth, and sixth years. Based on a previous study [17], we developed a questionnaire comprising three sections: Section A comprising gender and level; Section B containing 12 questions relating to the positive perception of online teaching; and Section C comprising 11 questions about the negative perception of online education (Appendix). Each question contained five answer choices (fully agree, agree, undecided, disagree, and fully disagree). The questionnaire was created on Google Drive (Google, Mountain View, CA) and circulated in the university’s pathology groups via WhatsApp and email. Ethical approval was obtained from the Biomedical Research Ethics Committee (HAPO-02-K-012-2022-09-1202).

Statistical analysis

The data were transferred to an Excel sheet (Microsoft Corporation, Redmond, WA) from the Google Drive form and processed using SPSS (Statistical Package for the Social Sciences, version 22.0; IBM Corp., Armonk, NY). The proportion of students with positive and negative perceptions was compared using the chi-square test. A p-value of <0.05 was regarded as statistically significant.

## Results

Of the 110 students approached for participating in the study, 77% (n = 85) responded to the questionnaire. The proportion of females was slightly higher than males: female students were 43 (50.6%) and male students were 42 (49.4%). There was an equal response (44.6%) regarding the online study of pathology for males and females. The total number of questions was 23 (12 for positive and 11 for negative perceptions). The mean response to positive perception questions was 14.02 ± 6.804, and the mean for negative perception questions was 12.72 ± 5.444. About half of the students responded that online learning was more flexible than on-site (physical) learning. Almost 70% of the students agreed that the Blackboard platform made learning easy. About two-thirds of the students decided that the pre-lecture video produced by the teachers beforehand made the pathology lectures easier to understand. About 41% disagreed when asked whether online lectures can substitute on-site interactive classes. Almost half of the students agreed that asking questions online was much easier than doing so during on-site classes. Nearly 60% of the students agreed that homework was quickly done online, and more than 60% reported that they were adapting well to the COVID-19 pandemic (Table 1).

**Table 1:** Frequency of students’ positive awareness about online teaching of pathology

S. No	Question	Strongly agree	Agree		Neutral	Disagree		Strongly disagree
1	I am satisfied with my online studying of pathology	20.5% (20)	24.1% (20)		20.5% (20)	15.7% (20)		19.3% (20)
	Total	44.6%			20.5%	44.6%		
2	Regarding time, online is more flexible than on-site learning	24.1% (20)	28.9% (24)		21.7% (18)	12.0% (10)		13.3% (11)
	Total	53.0%			21.70%	25.30%		
3	Blackboard made learning activities easy	28.9% (24)	39.8% (33)		21.7% (18)	4.8% (4)		4.8% (4)
	Total	68.7%			21.7%	9.6%		
4	Access to pathology content is easier when using pre-lecture video							
		16.9% (14)	18.1% (15)		22.9% (19)	25.3% (21)		16.9% (14)
	Total	34.9%			22.9%	42.2%		
5	It’s challenging for me to take duty online							
		21.7% (18)	33.7% (28)		16.9% (14)	18.1% (15)		9.6% (8)
	Total	55.4%			16.9%	27.7%		
6	I have adapted well to the new teaching and learning experience during the COVID-19 pandemic							
		6.0% (5)	19.3% (16)		27.7% (23)	33.7% (28)		13.3% (11)
	Total	25.3%			27.7%	47.0%		
7	Since on-site classes were canceled, how satisfied have you been with the support of the teaching staff?							
		15.7% (13)	31.3% (26)		28.9% (24)	14.5% (12)		9.6% (8)
	Total	47.0%			28.9%	24.1%		
8	Will you prefer in-person teaching this academic year after taking the vaccine?							
		15.7% (13)	31.3% (26)		28.9% (24)	12.0% (10)		12.0% (10)
	Total	47.0%			28.9%	24.1%		
9	My contact with teachers during the online study is greater than during the face-to-face study	31.3% (26)		19.3% (16)	20.5% (20)	18.1% (15)	10.8% (9)	
	Total	50.6%			20.5%	28.9%		
10	The transformation sparks me from on-site to online	9.6% (8)	28.9% (24)		27.7% (23)	24.1% (20)		9.6% (8)
	Total	38.6%			27.7%	33.7%		
11	I like to accomplish my duties online than on-site	16.9% (14)	36.1% (30)		25.3% (21)	14.5% (12)		7.2% (6)
	Total	53.0%			25.3%	21.7%		
12	COVID-19 has had no impact on my learning improvement and career	10.8% (9)	16.9% (14)		24.1% (20)	31.3% (26)		16.9% (14)
	Total	27.7%			24.1%	48.2%		

A significant proportion (>50%) of students found no difficulties communicating with teachers during the pandemic. Nearly two-thirds of the students reported that they were able to focus better during online teaching compared to on-site instruction. About 60% of the students were satisfied with the teacher’s support. Regarding negative perceptions, nearly half of the students agreed that social distancing disturbed their social and inter-colleague communications. Around 46 students found it difficult to separate domestic activities from learning ones, and 57% agreed that COVID-19 disrupted their learning improvement and career options. About 40% of students stated that the pandemic affected their health; more than half of the students feared contracting the disease during on-site practical sessions. About 50% of the students reported that the instability of the internet affected their academic performance (Table 2).

**Table 2:** Frequency of students’ negative awareness about online teaching of pathology

S. No.	Question	Fully agree	Agree	Neutral	Disagree	Fully disagree
1	Asking questions online is much easier than on-site					
		26.5% (22)	25.3% (21)	22.9% (19)	15.7% (13)	9.6% (8)
	Total	51.80%		22.90%	25.30%	
2	I found difficulties interacting with teachers online	9.6% (8)	15.7% (13)	25.3% (21)	32.5% (27)	16.9% (14)
	Total	25.30%		25.30%	49.40%	
3	Physical separation from university affected my life quality at the time of social distancing	19.3% (16)	31.3% (26)	27.7% (23)	15.7% (13)	6.0% (5)
	Total	50.60%		27.70%	21.70%	
4	Physical separation from colleagues affected my life quality at the time of social distancing	19.3% (16)	32.5% (27)	25.3% (21)	16.9% (14)	6.0% (5)
	Total	51.80%		25.30%	22.90%	
5	I found it very hard to isolate study activities from home duties	16.9% (14)	33.7% (28)	20.5% (17)	24.1% (20)	4.8% (4)
	Total	50.60%		20.50%	28.90%	
6	My well-being is influenced by the COVID-19 pandemic	19.3% (16)	22.9% (19)	22.9% (19)	26.5% (22)	8.4% (7)
	Total	42.20%		22.90%	34.90%	
7	I am stressed about almost being uncovered to COVID-19 amid lab training	15.7% (13)	36.1% (30)	20.5% (17)	18.1% (15)	9.6% (8)
	Total	51.80%		20.50%	27.70%	
8	This year my grades were affected by the COVID-19 pandemic	33.7% (28)	24.1% (20)	19.3% (16)	19.3% (16)	3.6% (3)
	Total	57.80%		19.30%	22.90%	
9	Poor strength of the internet at home affects my study	20.5% (17)	30.1% (25)	22.9% (19)	21.7% (18)	4.8% (4)
	Total	50.60%		22.90%	26.50%	
10	The poor strength of the teacher’s internet affects my study	10.8% (9)	24.1% (20)	38.6% (32)	21.7% (18)	4.8% (4)
	Total	34.90%		38.60%	26.50%	
11	Is it more difficult for me to focus during online teaching compared to onsite teaching during the COVID-19 pandemic?					
		21.7% (18)	36.1% (30)	30.1% (25)	9.6% (8)	2.4% (2)
	Total	57.80%		30.10%	12.00%	

Positive perceptions were categorized as low, medium, and high. There was a significant difference between female and male students in terms of positive perception of online learning (p < 0.001). Likewise, negative perceptions were also classified as low, medium, and high. However, the male group showed a greater negative perception of online teaching compared to the female group (p = 0.035). There was no significant difference between the second and third-year students regarding both positive and negative perceptions (Table 3).

**Table 3:** The relation between students’ sex and level and positive and negative perceptions of studying pathology online

Positive perception		Low	Medium	High	P-value
Sex	Male	14 (34.1%)	18 (43.9%)	9 (22.0%)	0.001
	Female	3 (7.1%)	15 (35.7%)	24 (57.1%)	
Level	Level 2	13 (23.2%)	24 (42.9%)	19 (33.9%)	0.284
	Level 3	4 (14.8%)	9 (33.3%)	14 (51.9%)	
Negative perception					
Sex	Male	3 (7.3%)	23 (56.1)	15 (36.6%)	0.035
	Female	11 (26.2%)	23 (54.8%)	8 (19.0%)	
Level	Level 2	8 (14.3%)	31 (55.4%)	17 (30.4%)	0.573
	Level 3	6 (22.2%)	15 (55.6%)	6 (22.2%)	

## Discussion

This study evaluated the positive and negative effects of the COVID-19 pandemic on the study of pathology via online learning. Like many faculties worldwide, we were forced to shift from on-site to online learning in response to the declaration of the COVID-19 pandemic on March 11, 2020 [18,19]. Umm Al-Qura University adopted the Blackboard network as an online teaching platform, allowing us to interact with students via live lectures, seminars, and tutorials. Through this study, we learned that the students highly accepted the blackboard platform as a tool for studying pathology online. Previous studies have also reported similar findings [20,21]. We also found that the videos made by the teacher and shared before the lectures helped improve the students’ understanding of pathology during the pandemic. In line with the study that reported that the use of videos, in addition to conversations between tutors and students, improved the outcomes of the lecture [22], our results also showed that the students adapted well to the pandemic following the initial panic state, most probably due to good knowledge of the protective measures and continuing evidence of a relatively lower risk of COVID-19 in the younger population [23,24]. Furthermore, in our study, the students were satisfied with the support given by the teachers during the pandemic. This is in sharp contrast to the findings of the study, which reported that teachers faced many difficulties in supporting students due to a lack of experience in online teaching [25]. A possible explanation for the superior satisfaction observed in our study could be the extensive training provided to the teachers by our university’s deanship of learning and distance education. Our students also reported that social distancing disturbed their social and interpersonal communications during the pandemic and hindered their peer-learning opportunities. Similar findings were described by two studies that reported high adverse effects on students’ attitudes toward learning, especially peer learning, during the COVID-19 pandemic [26,27]. An important finding in this study was that students found separating domestic activities from learning during the pandemic challenging since most of our students live with their families and participate in their expanded families’ daily activities. Almost two-thirds of students agreed that the pandemic adversely affected their education and career progress. A 2021 study by Aristeidou and Cross concluded that the COVID-19 pandemic could be deemed a career disturbance that significantly affected student education and career [28]. Notably, this study revealed that most students were stressed about being unprotected from COVID-19 amid lab training, similar to some previously conducted research [29,30]. We also found a significant difference between male and female students (p < 0.001) in their perception of online classes. Female students had a significantly positive perception of studying online pathology. This difference between male and female students may be attributed to the flexibility of online teaching and the ease of accessing the supportive studying material provided via the Blackboard platform. Another important thing is that our students did not live on campus owing to the cultural practices in this part of the Middle East. Consequently, the male students in this study tended to have a low perception of the online study compared to face-to-face teaching.

This study had some limitations. The sampling technique chosen for conducting this study was a non-random selected sample comprising the Al-Qunfudah Faculty of Medicine students, a model for all medical students in Saudi Arabia. However, this can limit the generalizability of the results, but somehow the culture of students and the infrastructure of medical faculties in Saudi Arabia are almost the same.

## Conclusions

The opportunity for online learning lit a path to study pathology virtually. Female students had a more excellent positive perception of online pathology teaching. Videos made by the teacher enhanced the understanding of the subject during the pandemic, especially when sent before the lectures. The students agreed that homework was quickly done online, and the students needed help to isolate study activities from home duties. Extensive training provided to teachers can significantly increase the support given to students during online teaching.

## Appendices

**Table 4:** Questionnaire

Section A						
Sex	Male			Female		
Age						
Section B	Positive awareness about online teaching of pathology					
Number	Question	Fully agree	Agree	Neutral	Disagree	Fully disagree
	I am satisfied with my online studying of pathology.					
	Regarding time, online is more flexible than on-site learning.					
	Blackboard made learning activities easy.					
	It is easier to ask questions on- line.					
	Access to pathology content is easier when using a pre-lecture video.					
	It’s challenging for me to take duty online.					
	I have adapted well to the new teaching and learning experience during the COVID-19 pandemic.					
	Since on-site classes were canceled, how satisfied have you been with the support of the teaching staff?					
	Asking questions online is much easier than on-site.					
	The transformation sparks me from on-site to online.					
	I like to accomplish my duties online than on-site.					
	COVID-19 has had no impact on my learning improvement and career.					
Section C	Negative awareness about online teaching of pathology					
Number	Question	Fully agree	Agree	Neutral	Disagree	Fully disagree
	Will you prefer in-person teaching this academic year after taking the vaccine?					
	I found difficulties interacting with teachers online.					
	Physical separation from university affected my life quality at the time of social distancing.					
	Physical separation from colleagues affected my life quality at the time of social distancing.					
	I found it very hard to isolate study activities from home duties.					
	My well-being is influenced by the COVID-19 pandemic.					
	I am stressed about almost being uncovered to COVID-19 amid lab training.					
	This year my grades were affected by the COVID-19 pandemic.					
	Poor strength of the internet at home affects my study.					
	The poor strength of the teacher’s internet affects my study.					
	Is it more difficult for me to focus during online teaching than onsite teaching during the COVID-19 pandemic?					
